# The time-course of single-word reading: Evidence from fast behavioral and brain responses

**DOI:** 10.1016/j.neuroimage.2012.01.061

**Published:** 2012-04-02

**Authors:** O. Hauk, C. Coutout, A. Holden, Y. Chen

**Affiliations:** a MRC Cognition and Brain Sciences Unit, Cambridge, UK; b Department of Experimental Psychology, University of Oxford, UK

**Keywords:** Visual word recognition, EEG, MEG, Lexical decision task, Semantic decision task, Go/NoGo paradigm

## Abstract

We usually feel that we understand a familiar word “immediately”. However, even basic aspects of the time-line of word recognition are still controversial. Different domains of research have still not converged on a coherent account. An integration of multiple sources of information would lead to more strongly constrained theoretical models, and help finding optimal measures when monitoring specific aspects of word recognition impairments in patient groups. In our multimodal approach – combining fast behavioral measures, ERPs and EEG/MEG source estimation – we provide converging evidence for the latencies of earliest lexical and semantic information retrieval in visual word recognition. Participants performed lexical and semantic decisions (LD, SD) in a Go/NoGo paradigm. We introduced eye-blink latencies as a dependent variable, in order to measure behavioral responses that are faster and less variable than traditional button presses. We found that the earliest behavioral responses distinguishing stimulus categories can occur around 310 ms. Ex-Gaussian analysis of behavioral responses did not reveal reliable differences between LD and SD. The earliest ERP differences between Go and NoGo conditions occurred around 160 ms for both LD and SD. Distributed source analysis of combined EEG/MEG data estimated neuronal generators for the lexicality effect around 200 ms in the left anterior middle temporal lobe. Thus, behavior and brain responses provide coherent evidence that the brain starts retrieving lexical and semantic information near-simultaneously within 200 ms of word onset. Our results support models of word recognition that assume a continuous accumulation of task-related information from the stimulus, which might be described by Bayesian principles.

## Highlights

► Behavioral eye blink responses distinguish word categories around 310 ms. ► ERP responses distinguish lexical and semantic word categories around 160 ms. ► Lexical and semantic word information are retrieved early and near-simultaneously. ► Lexicality effects around 200 ms are localized to the left mid-temporal lobe.

## Introduction

Reading is a skill that appears effortless and automatic to most of us. Many authors have highlighted the remarkable speed and ease with which skilled readers can recognize printed words. In natural reading, our brain has to rapidly 1) identify symbols (letters and words) that share many features with a large set of orthographic competitors; 2) link these symbols to representations of meaning (semantics) which are complex and shaped by years of learning and experience; 3) integrate information from a continuous flow of these symbols into concepts and messages. Metabolic neuroimaging methods (such as functional magnetic resonance imaging, fMRI) have inherently limited time resolution, and cannot answer questions about absolute or relative timing of brain processes. Behavioral and electrophysiological measures both provide timing information. But despite decades of research, even the basic questions “How early is the brain sensitive to lexical and semantic information?” or “Are lexical and semantic information retrieved at the same time, or at different stages?” are yet unanswered. We here use a multi-modal approach consisting of fast behavioral, ERP and combined EEG/MEG data to provide converging evidence for a time-line of word recognition.

Why should one care much about timing? Electrophysiological data are increasingly relevant for computational models of word recognition and other cognitive processes (Barber and Kutas, 2007; Braun et al., 2006; Garagnani et al., 2008; Philiastides et al., 2006). If we want to constrain a model of lexical access, for example, we need to know whether the signal we are analyzing reflects brain processes of lexical access itself, or follow-up processes related to decision making. The functional interpretation of brain activation crucially depends on timing information. For the same reason, timing has important implications for studies on clinical populations. In order to compare a particular cognitive process and its neuronal correlates between patient groups, we need to make sure that we look at the brain when this particular process is running — not too early or too late. This does not imply that later reflections of a process are meaningless. A late component distinguishing between meaningful or meaningless letter strings can indicate whether a non-responsive patient still processes lexico-semantic information (Friederici et al., 1999; Hagoort et al., 1996; Kotchoubey et al., 2002), but it might not reflect the information retrieval stage itself. Analyses of reaction time distributions have previously investigated how task or stimulus properties affect different stages of the decision process in laboratory experiments (Gomez et al., 2007; Ratcliff et al., 2004). However, they cannot reveal the absolute temporal structure of word recognition in the brain, because non-decision-related processes (such as early stimulus encoding and response output) are conflated with each other. Online measures of brain activation, such as EEG and MEG, have the potential to disentangle these processes between stimulus presentation and response (Hämäläinen et al., 1993; Picton et al., 1995).

Current literature contains very discrepant views on how to map the processes involved in word recognition onto brain responses. Most authors agree that word recognition is accomplished in overlapping or cascaded stages, and that the underlying processes can be interactive (e.g. Barber and Kutas, 2007; Dien, 2009; Grainger and Holcomb, 2009; Pulvermuller et al., 2009; Sereno et al., 2003). However, the time scale at which these processes operate, and the degree to which they overlap, is a matter of intense debate. A range of studies – arguably reflecting the dominant or traditional view – associates brain responses up to about 250 ms with orthographic processes, with lexico-semantic effects occurring after about 300 ms. For example, in the context of their bi-modal interactive activation model Grainger and Holcomb (2009) describe a succession of components that reflect analysis of sublexical orthographic units (~ 150 ms), mapping of orthographic information to whole-word representations (~ 250 ms), and lexical processing (325 ms), followed by different parts of the N400 component reflecting semantic and conceptual processing. This model is mainly supported by a large body of behavioral and ERP studies employing intra- and cross-modal priming at different stimulus onset asynchronies, including masked priming. Similarly, based on single-word MEG studies Pylkkanen and Marantz (2003) propose that brain activity between 150 and 200 ms reflects letter string processing, and between 300 and 400 ms lexical processing. These and other authors have provided strong evidence that the N400 component is dynamic, and can be decomposed into different subcomponents (Dien et al., 2010; Lau et al., 2008; Pylkkanen and Marantz, 2003). Nevertheless, it is common to consider the N400 as one component, and measure N400 effects within one time window (usually around 300–500 ms). For example, in a recent study Laszlo and Federmeier (2010) analyzed effects of several lexico-semantic variables on average amplitudes in the time window 250 to 450 ms. The fact that these variables produced effects in the same time window was taken as evidence that “semantic access takes place in parallel with stimulus recognition”.

However, there are an increasing number of studies that argue for earlier lexical and semantic processes, based on behavioral and electrophysiological data, which conclude that effects in the N400 time range reflect post-recognition processes. Sereno and Rayner (2003) pointed out that word frequency effects on fixation durations in reading, which on average take about 250 ms, are indicative of lexical access between 100 and 200 ms. Consistent with this hypothesis, they found word frequency effects on the N1 ERP component (132–192 ms) in a lexical decision task (Sereno et al., 1998). Word frequency effects in similar latency ranges have meanwhile been reported in several other studies, and are commonly interpreted as reflecting lexical processing (Assadollahi and Pulvermüller, 2003; Dambacher et al., 2006; Hauk and Pulvermüller, 2004a; Hauk et al., 2006a, 2006b; Palazova et al., 2011). This fits together with findings that orthographic variables, such as bigram frequency, affect the brain response around 100 ms, suggesting close succession or even overlap of orthographic and lexical processes (Hauk et al., 2006a, 2006b).

The situation is similarly complicated with respect to semantics. Originally, the N400 was described for violations of sentence semantics (Kutas and Hillyard, 1980). Many studies have since used the N400 component to study semantics at the single-word level, demonstrating effects of semantic priming (Bentin et al., 1985; Franklin et al., 2007), concreteness (Kounios and Holcomb, 1994), or semantic category (Kiefer, 2001; see Kutas and Federmeier (2000) for review). While these studies leave no doubt that the N400 is a marker for semantic processing, it is less clear whether it reflects processes of semantic information retrieval itself. Semantics is clearly a complex phenomenon, and cannot be reduced to just one or even a few variables. While there seems to be some consensus in the literature that lexical processing can be tapped into by measures of word frequency, measures of semantics are clearly more variable (e.g. McRae et al., 2005). In studies on picture naming, it has been shown that the type of semantic information required by the experimental task can affect the conclusion about whether phonology and semantics are processed serially or in parallel (Abdel Rahman and Sommer, 2003). Not surprisingly, reports for the earliest latencies of brain responses modulated by semantics in word recognition vary considerably, from the N400 latency range, to about 200 ms and before (e.g., action- and object-categories, Hauk and Pulvermüller, 2004b; Moscoso del Prado Martin et al., 2006; Pulvermüller et al., 1999). A recent ERP study reported different time courses for several types of semantic variables (e.g., visual and functional) within the first 200 ms (Amsel, 2011).

The main conclusion from this short overview is that the empirical data about the time course of lexical and semantic information retrieval are largely inconsistent, and have led to different conclusions. While there seems to be agreement that lexical and semantic processing must follow some initial orthographic analysis of the input, the estimates for the absolute and relative timing of lexical and semantic processes differ widely. There may be several reasons for this discrepancy. First of all, different authors may put their emphasis on different types of data. If, for example, one accepts the logic that frequency effects on fixation durations in natural reading reflect lexical access, then interpreting peak N400 amplitudes as reflecting lexical access is futile, in particular since most N400 onsets have been shown to occur after the end of fixation durations (Dimigen et al., 2011). Instead, one has to look for lexical effects at earlier latencies, as suggested by Sereno and Rayner (2003). If, on the other hand, one interprets these behavioral effects as some sort of “familiarity check” rather than lexical access, and puts more emphasis on well-established priming effects on standard ERP components, one may support the model of Grainger and Holcomb (Grainger and Holcomb, 2009). Another issue is the reliability of the evidence. Frequency effects on fixation durations are well-established, but clearly smaller than those on lexical decision times (Kliegl et al., 2006; Rayner, 2009; Staub et al., 2010). The above-mentioned ERP effects of lexical and semantic variables before 200 ms are subtle and still partly inconsistent across studies with respect to topographies and exact latencies. However, it would be wishful thinking to assume that the most important effects are always the biggest ones — big may be boring. If there is compelling evidence from different sources that the early effects are the theoretically relevant ones, then we should put our efforts into optimizing our methodology accordingly.

In the present study, we present data from two experiments to provide converging evidence from different sources of data for the latencies of the earliest lexical and semantic information retrieval in visual word recognition. These different sources are behavior, response-related brain responses, and stimulus-related brain responses. We applied a logic with respect to the correspondence between behavioral and brain responses that has been established in the domain of object categorization (Fabre-Thorpe et al., 2001; Johnson and Olshausen, 2005; Thorpe et al., 1996 and VanRullen and Thorpe, 2001). These authors employed a “Go/NoGo” task, in which participants were asked to respond to one stimulus category (e.g. faces, vehicles etc.), but refrain from responding to stimuli from other categories. In order to decide whether participants are able to distinguish the stimulus categories, one needs to consider both hit rate (i.e., correct responses) and false-alarm rate (i.e., incorrect responses). Somebody who is just guessing will have an equal proportion of hits and false-alarms. Somebody who can discriminate well will have a high hit rate but low false-alarm rate. The latter case would be reflected in a high positive d-prime value, a sensitivity index used in signal detection theory (e.g. Wickens, 2002). This analysis can be applied to multiple separate time bins, in order to determine the earliest behavioral responses that show a positive d-prime value. In the studies cited above, the earliest behavioral responses distinguishing between object categories occurred around 300 ms. This already provides an upper limit on the earliest latency by which category-relevant information is available. Obviously, the brain must have processed this information before the behavioral response. Thorpe and colleagues found a divergence between ERP curves for Go and NoGo trials around 150 ms, i.e., 150 ms before the earliest informative behavioral responses (Fabre-Thorpe et al., 2001; Johnson and Olshausen, 2005; Thorpe et al., 1996; VanRullen and Thorpe, 2001). The ERP difference between Go and NoGo trials can be considered as a “neuronal amplifier” — no matter how small the differences between stimulus categories are, the Go/NoGo difference provides an indicator for the latency beyond which the stimulus categories are processed differently. Assuming that the earliest differential brain responses correspond to the earliest differential behavioral responses, the latency difference between these two provides us with an estimate for the time required for response planning and execution of about 150 ms, which is also consistent with values obtained for the oculomotor latency, i.e., the time between initiation and execution of a voluntary saccade (Rayner, 1998; Rayner et al., 1983).

In the present study, we applied this strategy to word recognition. Our participants had to discriminate between words and pseudowords in a lexical and between living and non-living concepts in a semantic Go/NoNo task. Stimuli were matched among categories and tasks on relevant psycholinguistic dimensions. In Experiment 1, this paradigm was used with button presses in combination with event-related potentials (ERPs), in close analogy to the experiments by Thorpe and colleagues described above. In Experiment 2, we used combined EEG and MEG, and pioneered eye blinks as a novel way for the recording of behavioral responses. We expected that eye blinks would lead to faster behavioral responses because they require less movement energy than finger responses, have shorter nerve conduction delays, and participants feel a constant “readiness” to blink. Early behavioral responses distinguishing between stimulus categories would provide strong motivation for a more detailed investigation of early brain responses. Furthermore, we used our combined EEG and MEG data to estimate the neuronal generators of early word recognition processes over time.

## Materials and methods

### Experiment 1: ERPs and button presses

#### Participants

14 right-handed monolingual native speakers of British English were entered into the final analysis (7 females). Their mean age was 23 years (s.d. 5 years). All had normal or corrected-to-normal vision and reported no history of neurological illness or drug abuse. Handedness was determined according to a simplified version of Oldfield's handedness inventory (Oldfield, 1971), revealing a mean laterality quotient of 83 (s.d. 21). Informed consent was obtained from all subjects and they were paid for their participation. This study was approved by the Cambridge Psychology Research Ethics Committee.

#### Stimuli

Each stimulus category (Lexical Decision (LD): words, pseudowords; Semantic Decision (SD): living, non-living) contained 180 items (i.e., 360 per task). All words were English content words. Stimulus categories were matched on several relevant psycholinguistic variables, both within and across tasks where possible. In LD, words and pseudowords were matched for number of letters, bi-gram and tri-gram frequency as well as orthographic neighborhood size (Coltheart's N), based on information from the CELEX psycholinguistic database (Baayen et al., 1993). The matching result is presented in Table 1. In SD, living and non-living categories were matched for the same variables as in LD, plus word form and lemma frequency. Words in LD and SD were matched for all of the above-mentioned variables, except for a significant difference in orthographic neighborhood size between words in LD and the non-living category in SD. Pseudowords were created according to the orthographic and phonotactic rules of British English. Word lists in LD and SD did not overlap.

#### Procedure

The sequence of tasks was counterbalanced across subjects. Each participant could practice each task until they felt comfortable with it. In each of the three tasks, participants had to press a button with their right index finger to one stimulus category (“Go” trials, e.g. words in LD), but refrain from pressing the button in the other category (“NoGo” trials, e.g. pseudowords in LD). One set of subjects was assigned words (LD) and living (SD) for button presses, for the other set of participants this assignment was reversed, i.e., pseudowords and non-living were target categories. Each stimulus was presented (black on white background, 18 point lower case Courier New font) for 100 ms. The stimulus onset asynchrony varied between 2350 and 2750 ms (mean 2.5 s). A small fixation cross was shown in the center of the screen when no stimulus was present. Stimulus delivery and response collection were controlled by E-Prime software (version 2, Psychology Software Tools Inc., Pittsburgh, USA). The button box was provided by Berisoft (http://www.berisoft.com/). Every participant received a different randomization of stimuli. Participants were instructed to minimize eye and body movements throughout the experiment.

#### Data recording

The electroencephalogram (EEG) was measured in an electrically and acoustically shielded EEG chamber at the MRC Cognition and Brain Sciences Unit in Cambridge, UK. Data were recorded from 65 Ag/AgCl electrodes, all of which were mounted on an electrode cap (EasyCap, Falk Minow Services, Herrsching-Breitbrunn, Germany) except the lower vertical EOG electrode which was placed below the right eye, using SynAmps amplifiers (NeuroScan Labs, Sterling, USA). Electrodes were arranged according to the extended 10/20 system. Data were sampled at 500 Hz with a band-pass filter 0.1–100 Hz. Cz was used as recording reference for the EEG channels. The EOG was recorded bipolarly through electrodes placed above and below the left eye (vertical) and at the outer canthi (horizontal).

#### Pre-processing of ERP data

The continuously recorded data were band-pass filtered between 1 and 40 Hz. For averaging, they were divided into epochs of 500 ms length, starting 100 ms before stimulus onset. Trials with peak-to-peak potential differences larger than 100 μV in EEG or EOG channels were rejected, as were trials in which incorrect responses were given. For each channel the mean amplitude of a 100 ms baseline interval was subtracted at all time points, and data were converted to average reference.

#### Analysis of behavioral data

Response times (hits in Go trials and false-alarms in NoGo trials) were organized in successive time bins of 25 ms duration for each participant individually. D-prime values for hits and false-alarms were computed for each time bin, and these were averaged across participants (Fig. 2). Of particular interest is the earliest in a sequence of time bins with positive d-prime values. The onset of the distribution is the first detectable signal above noise level, and therefore the effects are of small magnitude. The logic of the analysis is that we start with an effect that is undoubtedly significant (i.e., the clearly positive d-primes around the mean response time), and work our way back to the first positive d-prime above baseline level. We defined the onset of the positive d-prime distribution as the first latency before the peak at which the d-prime value exceeded the standard deviation of d-prime values in the interval from 0 to 300 ms by a factor of at least 2. As shall be described in the following, we also fitted Ex-Gaussian distributions to our data, in order to compare reaction time distributions between LD and SD tasks statistically.

**Fig. 1:**
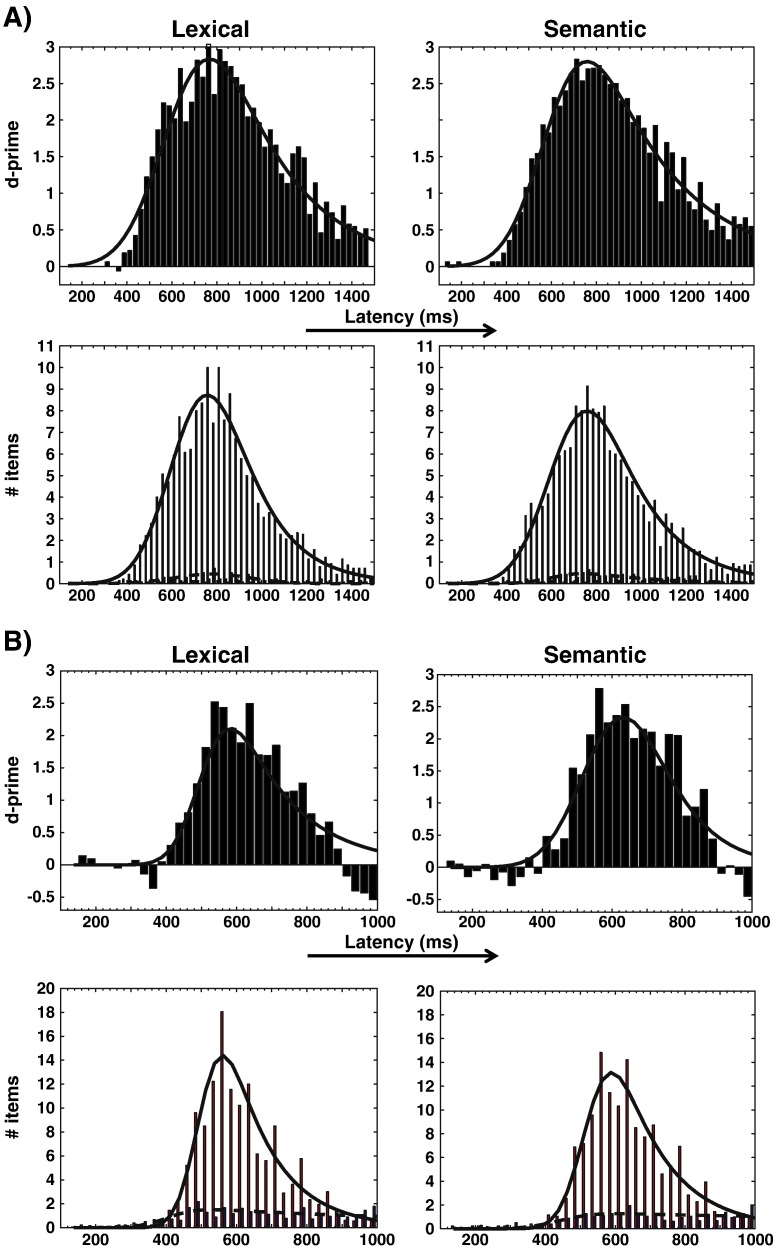
Reaction time histograms for time bins of 25 ms duration and their corresponding d-prime values in two Go/NoGo experiments (means across participants). A) Button press lexical decision latencies from Experiment 1. Positive d-primes indicate that the corresponding response time bin contains more hits (red) than false-alarms (blue). The superimposed curves represent Ex-Gaussian functions fitted to histograms for hits and false alarms separately. B) As in A), but derived from peak latencies for eye blinks in the electrooculogram in Experiment 2.

In many studies, behavioral data are summarized by averaging across items and/or participants. Although standard deviations or standard errors are commonly reported, in general only average response times are subjected to statistical analysis as dependent variables. This procedure is efficient, but may ignore valuable information in the distribution of response times. Furthermore, the use of mean and standard deviation implicitly assumes sampling from a Gaussian distribution. Reaction time distributions, however, are usually skewed towards larger values, as in our data (see Fig. 1). This type of distribution can be summarized more accurately by a set of three parameters using the Ex-Gaussian distribution (Matzke and Wagenmakers, 2009). The Ex-Gaussian distribution is a convolution of a Gaussian and an Exponential function (Lacouture and Cousineau, 2008). These are described by three parameters: mu and sigma, which are related to the mean and standard deviations of the Gaussian component, respectively; and tau, which represents the mean of the exponential component (Matzke and Wagenmakers, 2009):

$$f(x|\mu ,\sigma ,\tau )=\frac{1}{\tau \sqrt{2\pi }}\exp\left(\frac{\sigma^{2}}{2\tau^{2}}-\frac{x-\mu }{\tau }\right)\int_{-\infty }^{\left[(x-\mu )/\sigma ]-(\sigma /\tau \right)}\exp\left(-\frac{y^{2}}{2}\right)dy\text{.}$$

Note that the mu and sigma are not numerically the same as the mean and standard deviation in a conventional analysis, which would not include the exponential component. Although the three parameters cannot be interpreted in terms of specific cognitive processes (Matzke and Wagenmakers, 2009), they allow a more detailed description of behavioral data than would be possible using the mean alone. For example, an effect only on the exponential component (i.e., the “tail” of the distribution) may be indicative that an experimental manipulation only affects late, and not all, responses (Staub et al., 2010).

We used the three Ex-Gaussian parameters to compare behavioral results between LD and SD tasks. Ex-Gaussian distributions were fitted to d-prime distributions computed for reaction time bins as described above, for individual participants and conditions separately. We then compared Ex-Gaussian parameters between tasks using paired two-tailed t-tests. In addition, we derived estimates for the onsets of positive d-prime distributions for individual participants. D-prime distributions at the single-subject level are based on a relatively small number of samples, and determining their onsets based on the analysis of separate histogram bins is therefore unreliable. Instead, we fitted Ex-Gaussian functions to these distributions, and derived a measure for onset latencies from those in the following manner: In brief, we fitted a line to the rising slope of the Ex-Gaussian functions, and defined the intersection of this line with the x-axis as the onset of the distribution. In order to do so, we first determined the point of maximum slope in the rising phase of the Ex-Gaussian function. This was done by determining the maximum value of its first derivative. We then fitted a line with the slope of the Ex-Gaussian at this point, i.e., the value of its first derivative. The point where this line crossed the x-axis was taken as the onset of the distribution. We would like to point out that the onset of a distribution is – by definition – the smallest detectable deflection from baseline, and therefore inevitably less reliable to determine from empirical data than for example its peak. The Ex-Gaussian function generally provides a good fit to reaction time distributions, but may not accurately capture all of their aspects, such as their onsets. We therefore used the values derived from our Ex-Gaussian analysis for statistical comparison of LD and SD tasks, but the estimates for the onset latencies used in our discussion are based on the first positive d-prime values in Fig. 1, as described above. As it turned out, these values were more conservative than those based on Ex-Gaussians. We used partly modified functions from the Matlab toolbox of Lacouture and Cousineau (2008) to fit Ex-Gaussians to our behavioral data. Because the Ex-Gaussian cannot be negative, any negative d-prime values in our data were set to zero before fitting.

#### Statistical analysis and display of ERP data

Time courses of ERP data are displayed in two ways:

1) as root-mean-square curves across all electrodes $\left(\sqrt{\frac{1}{63}\sum_{i=1}^{63}x_{i}^{2}}\right)$;
2) as average voltages within 7 separate electrode groups, each group representing electrodes in both hemispheres along a line at a different anterior–posterior position, namely frontal (F1, F3, F5, F7, Fz, F2, F4, F6, F8), fronto-central (FC1, FC3, FC5, FT7, FT9, FCz, FC2, FC4, FC6, FT8, FT10), central (C1, C3, C5, T7, Cz, C2, C4, C6, T8), centro-parietal (CP1, CP3, CP5, TP7, TP9, CPz, CP2, CP4, CP6, TP8, TP10), parietal (P1, P3, P5, P7, Pz, P2, P4, P6, P8), parieto-occipital (PO3, PO9, POz, PO4, PO10), and occipital (O1, Oz, Iz, O2). This is analogous to the presentation of ERP results in VanRullen and Thorpe (2001).

Statistical analysis was performed using the “SensorSPM” method implemented in SPM5 (http://www.fil.ion.ucl.ac.uk/spm/; http://imaging.mrc-cbu.cam.ac.uk/meg/SensorSpm). This method applies Random Field Theory (RFT), originally developed for the analysis of three-dimensional fMRI activation patterns (Worsley, 2003), to EEG data. The EEG electrodes are represented along two spatial dimensions (x and y), and the time course is represented as a third dimension (z) (Kiebel and Friston, 2004a). For this purpose, the EEG data were linearly interpolated from their original electrode locations to a 32 × 32 grid. The data were additionally smoothed using a smoothing kernel with dimensions 5 mm (x and y) and 10 ms (z), to make them more compatible with the assumptions of random-field theory. The whole data set is therefore represented as a three-dimensional volume, which allows application of RFT in the same manner as for fMRI data. Importantly, RFT accounts for the multiple comparisons problem in space and time, by estimating the number of degrees of freedom from the data (Kiebel and Friston, 2004b). This does not require the pre-selection of electrodes or time windows. As will be specified below, results will be displayed at a significance threshold of 0.05 corrected either for family-wise errors (FWE) or for false discovery rate (FDR) (Benjamini and Yekutieli, 2001). Statistics at the voxel- and cluster-level were obtained from the non-stationarity toolbox in SPM5 (Hayasaka et al., 2004).

The output of the SPM analysis consists both of significance values for clusters and voxels. A voxel in this context is a data point defined by location and latency, e.g., electrode Cz at latency 200 ms. A cluster consists of voxels that are significant and adjacent to each other in space and time, i.e., either electrodes that are close to each other at the same latency, or successive time points for the same electrode. This method does not test for topographical differences between conditions (e.g., reflected in an interaction with a factor “Electrode”). However, our predictions are about generally larger amplitudes in the Go compared to the NoGo conditions, for which this mass uni-variate approach is well-suitable.

The advantage of the SensorSPM approach is that it does not rely on a priori defined time windows or electrode groups, e.g., based on peak latencies or electrodes. It allows for variability of effects in space and time while controlling for multiple comparisons. This is of particular relevance for effects that cannot reasonably be assumed to correspond to standard “components” — there is no logical reason why effects of subtle experimental manipulations should occur around the peaks of the basic evoked potential or field. Furthermore, effects may vary across experiments due to a number of factors, which may be difficult to account for with a rigid choice of time windows. SensorSPMs are therefore arguably more objective and standardized than traditional time window analyses, at least in situations where experimental effects do not follow a standard component logic. This comes at a cost — SensorSPMs are more difficult to interpret and to visualize. The results are not reduced to one or a few significance values, but rather provide statistics for voxels and clusters in a three-dimensional spatio-temporal grid. In our study, we were interested in the earliest latencies at which the brain distinguished between Go and NoGo trials. Based on the literature and our behavioral results, we predicted this to occur before 200 ms, but could not specify a particular component or time window. We therefore defined a time window for SensorSPM analysis between 100 and 400 ms, which comprised our latency range of interest as well as later latencies for which effects can be expected. We included all 63 EEG electrodes into the following analyses.

### Experiment 2: EEG + MEG and eye-blinks

#### Participants, stimuli and procedure

The stimuli and general procedure were the same as in Experiment 1, except that participants had to respond using eye blinks rather than button presses. 18 right-handed monolingual native speakers of British English were entered into the final analysis (8 female). Their mean age was 23 years (s.d. 5 years). All had normal or corrected-to-normal vision and reported no history of neurological illness or drug abuse. Handedness was determined according to a simplified version of Oldfield's handedness inventory (Oldfield, 1971), revealing a mean laterality quotient of 92 (s.d. 11). Informed consent was obtained from all subjects and they were paid for their participation. Stimuli were presented with 100 ms duration and an SOA between 2050 and 2650 ms (18 point lower case Courier New font, white on black). We noticed in pilot studies that some participants find it hard to restrain from blinking during the whole trial. We therefore presented the word “end” in red letters 1000 ms after stimulus onset, indicating that they could blink at this point no matter which stimulus had appeared on the screen. Although this procedure differed from Experiment 1, we did not provide any feedback about correct or incorrect responses, and did not change the instructions with respect to speed and accuracy. The reaction time distributions in Fig. 1 demonstrate that the 1000 ms period was enough to capture also the tail of the distribution. This study was approved by the Cambridge Psychology Research Ethics Committee.

#### Recording procedure

The magnetoencephalogram (MEG) was measured in a magnetically and acoustically shielded MEG booth at the MRC Cognition and Brain Sciences Unit in Cambridge, UK. The MEG was recorded with the 306-channel Neuromag Vectorview system (Elekta AB, Stockholm) combining 204 planar gradiometers and 102 magnetometers at a sampling rate of 1000 Hz. The position of 5 Head Position Indicator (HPI) coils attached to the EEG cap, 3 anatomical landmark points (nasion and preauricular points), as well as 50–100 additional randomly distributed points (head shape), were digitized with a 3Space Isotrak II System for an accurate coregistration with MRI data. The electrooculogram (EOG) was recorded bipolarly through electrodes placed above and below the left eye (vertical EOG) and at the outer canthi (horizontal EOG).

#### Pre-processing

In a first step, the signal-space separation (SSS) method implemented in the Neuromag “Maxfilter” software was applied to our MEG data, in order to remove artifacts likely to arise from sources distant to the sensor array (Taulu and Simola, 2006). In this process, the spatio-temporal variant of SSS as well as movement compensation was applied, and statically bad channels were interpolated. Data were inspected visually, and EEG channels considered to be faulty were excluded from further analysis. Data were then band-pass filtered between 0.1 and 40 Hz using the MNE software. Data were divided into epochs of 600 ms length, starting 100 ms before stimulus onset. Epochs were rejected when maximum–minimum amplitudes exceeded the following thresholds in the interval − 100 to 100 ms around stimulus onset: 100 μV in EOG and EEG, 2000 fT in magnetometers, and 1000 fT/cm for gradiometers. Latencies after 100 ms were not taken into account because they may have contained eye blink responses required by the task. For each channel, the mean amplitude of a 100 ms baseline interval was subtracted at all time points.

Peak latencies for eye blinks were determined in each trial as the latency of maximum voltage in the vertical EOG channel between 200 and 1000 ms, if the amplitude around the peak stayed above 80 μV for at least +/− 50 ms, i.e., a 100 ms interval around the peak. In Go trials, such a response was counted as a hit, in NoGo trials as a false positive. If no such peak could be determined, it was considered a miss or correct rejection, respectively. Only epochs corresponding to hits or correct rejections were averaged. We used peak latency as dependent variable in our behavioral analyses. However, it is important to note that the earliest behavioral response is the onset of the blink, i.e., the beginning of the movement of the eye lid. For example, the average down phase duration of spontaneous eye blinks has been estimated to be 92 ms (VanderWerf et al., 2003). We will take this into account in the interpretation of our results.

#### Source estimation and statistics

We used the MNE software package (Version 2.6; http://www.nmr.mgh.harvard.edu/martinos/userInfo/data/sofMNE.php) in combination with FreeSurfer (Version 4.3.0; http://surfer.nmr.mgh.harvard.edu/) in order to apply minimum norm source estimation to our combined EEG and MEG data. The noise covariance matrices for each data set were computed for baseline intervals of 200 ms duration before the experimental stimuli. In the standard MNE procedure, it is used to transform the measurements into signal-to-noise ratios (SNRs), such that data from different sensor types (magnetometers, gradiometers and electrodes) can be combined for source estimation. The default regularization procedure implemented in MNE was used. MEG sensor configurations and MRI images were coregistered based on the matching of about 50–100 digitized locations on the scalp surface with the reconstructed scalp surface from the FreeSurfer software (see below).

High-resolution structural T1-weighted MRI images were acquired in a 3 T Siemens Tim Trio scanner at the CBU using a 3D MPRAGE sequence, field-of-view 256 mm × 240 mm × 160 mm, matrix dimensions 256 × 240 × 160, 1 mm isotropic resolution, TR = 2250 ms, TI = 900 ms, TE = 2.99 ms, flip angle 9°. Structural MRI images were processed using automated segmentation algorithms of the FreeSurfer software (Dale et al., 1999; Fischl et al., 1999). The original triangulated cortical surface (consisting of several hundred thousand vertices) was down-sampled to a grid using the traditional method for cortical surface decimation with an average distance between vertices of 5 mm, which resulted in approximately 10,000 vertices. A boundary element model (BEM) was created using a watershed algorithm, containing 5120 triangles for the inner skull surface, outer skull surface and skin surface. Dipole sources were assumed to be perpendicular to the cortical surface. Source estimates were computed for each subject and predictor variable. The individual results were morphed to the average brain across all subjects, and a grand-average was computed. These grand-averages were then displayed on the inflated average cortical surface.

Regions-of-interest (ROIs) were defined based on the source estimates for the average across all stimuli. We determined peaks of activation that were activated within the first 200 ms, and fell within areas of interest. The borders of these ROIs were defined manually using the “mne_analyze” function in the MNE software package, and followed approximately the line of half-peak-amplitude around a peak. Note that comparisons of amplitudes between ROIs are usually not informative, because they may simply reflect different sensitivities of the sensor configuration to different brain areas.

In order to label the peaks in our source estimation results, we obtained their Talairach coordinates in the FreeSurfer software, and submitted them to the “Talairach Daemon” (University of Texas Health Science Center San Antonio, http://www.talairach.org/daemon.html). We would like to point out that this was done for reasons of standardization and objectivity, rather than for a direct comparison with other neuroimaging modalities such as fMRI. The results have to be interpreted with respect to the general resolution limits of EEG/MEG measurements (Hauk et al., 2011; Molins et al., 2008).

## Results

### Behavior

Fig. 1 presents reaction time distributions of Experiments 1 (Fig. 1A, button presses) and 2 (Fig. 1B, eye blinks). Bar graphs for histograms were computed for time bins of 25 ms width. We superimposed fitted Ex-Gaussian functions for hits (red bars), false-alarms (blue) and d-prime (black) values, respectively. Fig. 1A demonstrates that hit rates for button presses exceeded false-alarm rates already for responses around 375 ms, as reflected by positive d-prime values. For peak eye-blink latencies (Fig. 1B), the onset of the positive d-prime distribution was at 400 ms. We attempted to corroborate these findings using estimates for the onsets of positive d-prime distributions based on an Ex-Gaussian analysis. Onsets for individual participants and conditions were estimated based on the points of maximum slope for the fitted Ex-Gaussians. In Experiment 1, the mean estimate across subjects for LD was 422 (s.d. 79) ms and for SD 430 (s.d. 85) ms. In Experiment 2, these values were 343 (s.d. 164) ms and 363 (s.d. 177) ms, respectively. These estimates confirm that behavioral responses around or even before 400 ms are sensitive to stimulus categories. However, since we cannot be sure how well Ex-Gaussians capture the onsets of reaction time distributions, we will use the estimates derived from Fig. 1 in our discussion, which for the earliest eye blink latencies in Experiment 2 are more conservative.

The average latency between peak latency and blink onset has been estimated to be around 92 ms (VanderWerf et al., 2003), which means that the earliest behavioral responses (movement of the eye lid) distinguishing word categories must have occurred around 310 ms. These results already provide an upper limit for the earliest latency by which lexico-semantic information is available in the brain — if behavior is sensitive to this kind of information around 310 ms, then the brain must have processed it even before. Subtracting an estimated 150 ms from the latencies reported above (Rayner, 1998; Thorpe et al., 1996), we arrive at 225 ms (button press) and 160 ms (eye blinks), respectively, for the latency at which lexico-semantic information should affect the brain response.

**Fig. 2:**
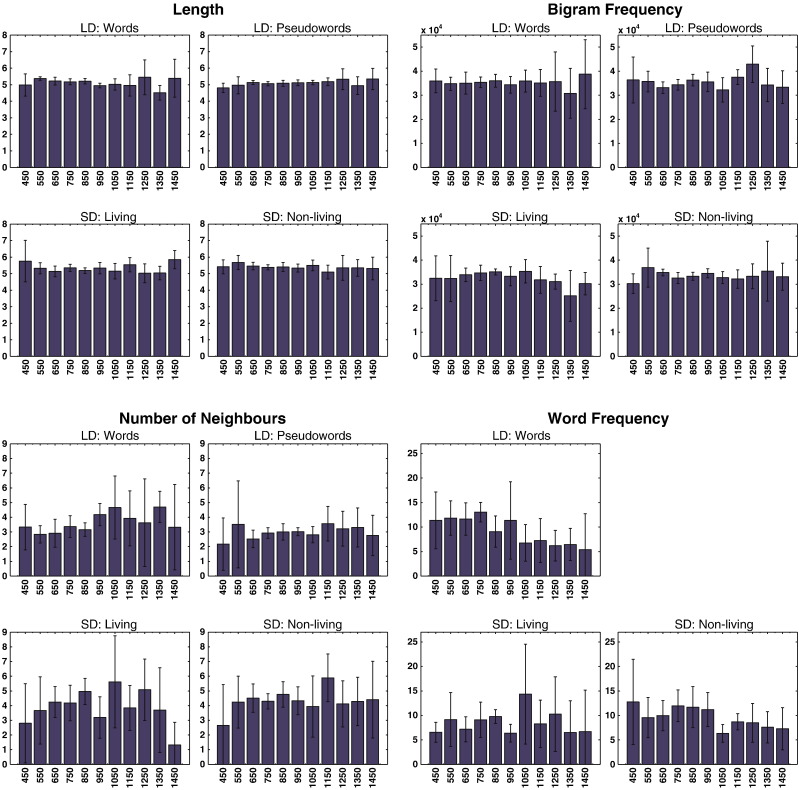
Histogram plots for reaction time data of Experiment 1. For time bins of 100 ms duration, centered around latencies 450 to 1450 ms in 100 ms steps, average values of several psycholinguistic variables were computed for those items that were responded to within these latency bins. This allows testing whether the earliest responses shared particular psycholinguistic features (and complements Table 1). Error bars are standard deviations. Frequency values are reported as per million. Note that there are no word frequency measures for pseudowords. LD: lexical decision; SD: semantic decision.

It is possible that the earliest button presses or eye-blinks are triggered by the same small set of stimuli with untypically salient features. Our stimulus selection and matching already renders this unlikely, since we did not use words of extremely high frequency or with other untypical properties (e.g., no function words such as “the”). In order to address this problem more directly, we sorted our items for each individual participant with respect to response speed. Tables 2 and 3 present the items for which participants responded fastest. These items are different across subjects, and do not suggest any obvious pattern. This is complemented by Fig. 2, which shows histrogram plots for several important psycholinguistic variables, such as word length, frequency etc. The earliest responses do not stand out with respect to any of these variables, which can therefore not have confounded our results.

In Experiment 1, mean button press latencies for hits were numerically smaller for LD than SD (850 vs 871 ms, s.d. 107 vs 129 ms, respectively), as would be expected if semantic information retrieval took longer than lexical information retrieval, but this difference was not significant (t(13) = − 0.84, p > 0.4). Error rates were 4.4% and 5.4%, respectively, and the difference was not significant (t(13) = 0.24). In Experiment 2, mean blink peak latencies for hits significantly differed between LD and SD with values of 621 (s.d. 73) ms and 647 (s.d. 57) ms, respectively (t(17) = − 2.97, p < 0.01; from EOG). The rejection rates for trials were 23% in both LD and SD, which did not differ significantly (t(17) = − 0.01). Note that this included both behavioral errors and rejection due to artifacts.

The onset latencies of positive d-prime values obtained above were the same in LD and SD tasks. We therefore analyzed the corresponding RT distributions in more detail. We fitted Ex-Gaussian functions to RT distributions of hit responses. None of the corresponding comparisons was significant in neither experiment, indicating that the two distributions do not differ significantly from each other. In Experiment 1, the average Ex-Gaussian parameters were *μ* = 655 (s.d. 87), *σ* = 114 (32), *τ* = 195 (59) for LD, and *μ* = 658 (80), *σ* = 123 (31), *τ* = 213 (65) for SD. In Experiment 2, these values were *μ* = 556 (s.d. 90), *σ* = 74 (48), *τ* = 65 (43) for LD, and *μ* = 567 (81), *σ* = 69 (49), *τ* = 80 (45) for SD.

### ERP results

**Fig. 3:**
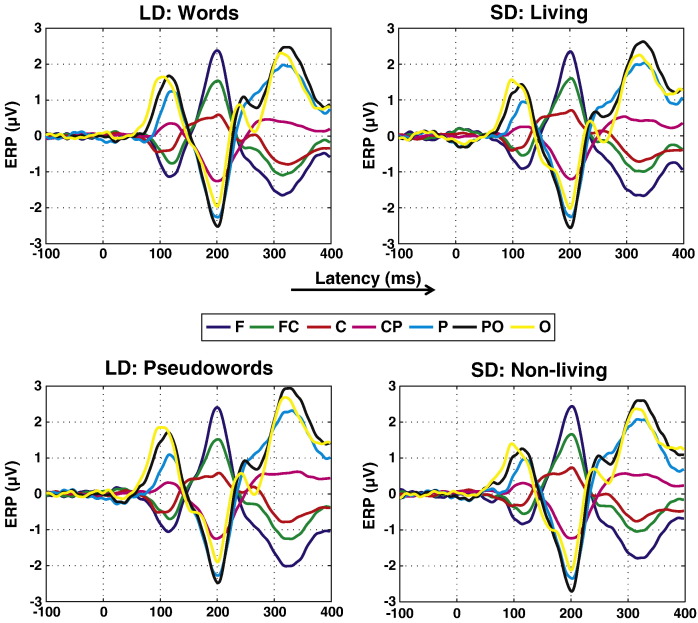
ERPs from Experiment 1 for each individual stimulus category. Individual curves correspond to mean voltages within electrode groups. F: frontal; C: central; P: parietal; O: occipital. LD: lexical decision; SD: semantic decision.

The ERP curves for data averaged within the individual conditions of Experiment 1, i.e., words and pseudowords in lexical decision and living and non-living in semantic decision, are presented in Fig. 3. Each curve represents an electrode group from anterior to posterior, as indicated by the color-coding. As expected, the pattern of results is very similar across the different conditions.

**Fig. 4:**
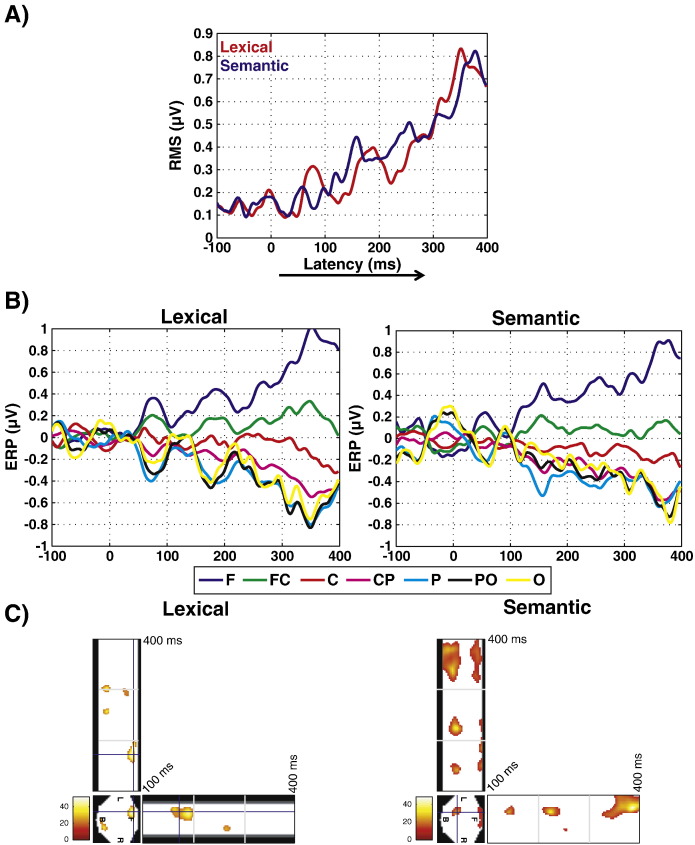
Difference ERPs from Experiment 1, displayed in the same way as original ERPs in Fig. 3. A) Time-course of overall signal strength computed using the root-mean-square (RMS) signal across all electrodes, for lexical and semantic decision tasks (LD and SD) separately. B) ERPs over time for separate electrode groups (anterior to posterior). F: frontal; C: central; P: parietal; O: occipital. Results for LD and SD are displayed separately. C) SensorSPMs, displaying F-value topographies over time corresponding to the data shown in A) and B). Red-to-yellow color indicates time ranges where F-values exceed the FDR-corrected threshold for significance taking into account multiple comparisons across electrodes and time points. The square at the bottom-left of each panel represents the electrode array unfolded onto a plane surface (F/B/L/R: front/back/left/right), and F-values at the peak of the earliest significant cluster. The horizontal panels show the time course of F-values for the lines dissecting the electrode array vertically; the vertical panels for the lines dissecting it horizontally. Note that this represents only a cross-section of the 3-dimensional (space-x-time) data.

From our behavioral results we predicted that ERP difference responses for Go minus NoGo conditions should diverge from zero around 150–200 ms. The corresponding ERP data from Experiment 1 are presented in Fig. 4. Fig. 4A presents the Root-Mean-Square (RMS). The peak of this measure of overall signal strength occurred between 300 and 400 ms in both the lexical and semantic decision task. However, the signal already started to diverge from baseline before 200 ms. Fig. 4B presents these data in more detail, separated into electrode groups from anterior to posterior areas. The pattern of results is very similar to that presented in previous studies on object recognition (e.g. VanRullen and Thorpe, 2001). The anterior positivity for the Go–NoGo difference is commonly associated with response inhibition in the NoGo condition (Muller and Hagoort, 2006; Simson et al., 1977; Thorpe et al., 1996). We would like to point out that the origin or generators of this response is not crucial for our conclusions — any early behavioral or brain response that distinguishes between stimulus categories poses constraints on the preceding processes.

The ERP results were confirmed statistically by SensorSPM analysis, taking into account multiple comparisons across space and time. Our analysis consisted of two stages. First, we combined both LD and SD into one analysis, testing whether there were consistent Go/NoGo differences across both tasks. This analysis used family-wise error (FWE) correction for multiple comparisons. Second, we tested for Go/NoGo difference for each task separately, using the less conservative false discovery rate (FDR) criterion. We also performed this second analysis on data in the latency range − 100 to 100 ms, but did not find any significant effects. Our results are therefore unlikely to represent baseline fluctuations. The following effects were significant both at voxel- and cluster-level.

For the combined analysis, the earliest significant effects at a FWE-corrected threshold occurred at 160 ms (F(27) = 31.31, cluster size k = 30) and 190 ms (F(27) = 35.82, k = 151). The largest F-value was obtained at 362 ms (F(27) = 39.83, k = 521). For the lexical decision task alone, we found the earliest significant differences at an FDR-corrected threshold between Go and NoGo conditions at 168 ms (F(13) = 47.17, local maximum of a cluster with main peak at 186 ms, F(13) = 51.60, k = 590). The largest F-value in the lexical decision task occurred at 186 ms (above), followed by an effect around 305 ms (F(13) = 44.27, k = 311). The earliest effects in the semantic decision task were obtained at 166 ms (F(13) = 28.15, k = 1795), with the largest F-value at 338 ms (F(13) = 48.08, k = 4143). These results present strong evidence that Go and NoGo trials produce different brain responses already before 200 ms after stimulus onset, although the strongest responses occurred at later latencies after 300 ms. This corresponds to the ERP curves presented in Figs. 3A and B.

We would like to note that visual inspection of the ERP curves for the lexical decision task in Fig. 4B appears to show a deflection from baseline already just before 100 ms. We did not predict an effect at this early latency, and as noted above, it did not exceed the significance threshold in our SensorSPM analysis. This deflection around 100 ms is only present in the lexical decision task. Differences between words and pseudowords have been reported at such early latencies in the literature (Hauk et al., 2009; Segalowitz and Zheng, 2009), and could therefore potentially contribute to the earliest differences between Go and NoGo trials. In the object recognition literature, there have recently been reports of ultra-rapid categorization around 100 ms (Kirchner and Thorpe, 2006). At this point, we conclude that our statistical analysis does not support such an ultra-rapid categorization for words, but this should be investigated in more detail in future studies.

### EEG/MEG source estimation

**Fig. 5:**
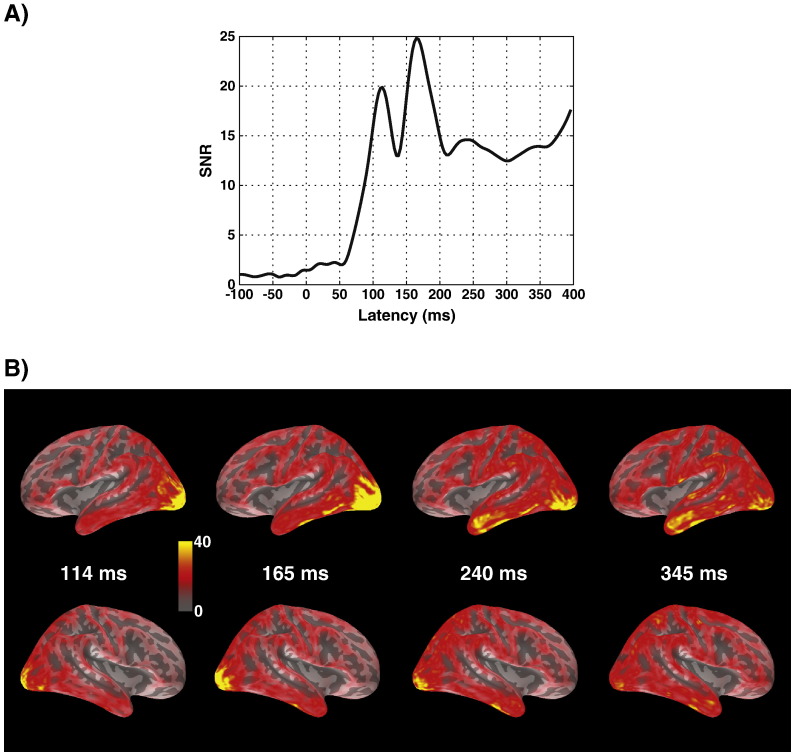
Time course of EEG/MEG data and source estimates in Experiment 2. A) Time course of signal-to-noise ratio for the combined EEG and MEG data. B) Minimum norm source estimates for the average of words and pseudowords at peak latencies derived from A), viewed from the left (top) and right (bottom). Units are in pA.

Fig. 5 illustrates the general time course of the EEG/MEG signals and their neuronal sources. Fig. 5A shows the root-mean-square (RMS) of signal-to-noise ratio (SNR) of the EEG/MEG response averaged across words and pseudowords over time. SNRs were computed for individual channels first, by dividing the signal at every time point by the standard deviation of the baseline interval. This renders the signals unitless, and allows averaging the values across different sensor types (the original physical measurement units are V, T, and T/m, respectively, and cannot be combined in simple averaging). The result shows that average SNRs at the P1 and N1 peaks are about 20 and 25, respectively, and stay around 15 afterwards.

Fig. 5B presents minimum norm source estimates from combined EEG/MEG data for the average of words and pseudowords on an average inflated cortical surface at peak latencies derived from the curve in Fig. 4A. At 114 ms, activity is clearly centered around the occipital lobe, with maximum in left occipital gyrus. At 165 ms, it has spread forward along the inferior temporal gyrus, mostly in the left but to a smaller degree also in the right hemisphere. By 240 ms, peaks of activation occur in the left anterior temporal lobe. The pattern of activation around 240 ms appears to be stable until about 345 ms. Later latencies were already contaminated by eye blink activation, and therefore not suitable for source estimation. Our results are generally consistent with a spread of activation from posterior to anterior areas within about 300 ms as reported in previous studies (Dhond et al., 2007; Marinkovic et al., 2003).

If the brain already initiates decision- or response-related processes around 200 ms, as suggested by our ERP results, then it must also have processed stimulus-specific information around this latency. Our Go/NoGo results from Experiment 1 suggest that the brain should be sensitive to lexicality already around 160 ms. However, the earliest effects may not necessarily have a sufficiently high signal-to-noise ratio to reach significance in the source estimates. A previous ERP study on lexicality effects (and their relationship to orthographic typicality) found a main effect of lexicality around 200 ms (Hauk et al., 2006a, 2006b). We therefore chose a time window around 200 ms (180–220 ms) for statistical analysis in source space. The localization of lexicality effects at this latency is less clear. The aforementioned study, based on minimum-norm estimates applied to grand-mean ERP data, found the major sources in an area compatible with the left anterior temporal lobe. A study using similar methodology localized effects of lexical word frequency to the left temporal lobe as well, albeit more posteriorly (Hauk et al., 2006a, 2006b). Both middle and anterior temporal lobes of the left hemisphere have been implicated in linking word forms with semantic networks (e.g. Patterson et al., 2007; Price and Mechelli, 2005). Anterior mid-temporal cortex has also been suggested as a putative generator of the N400 (Dobel et al., 2010; Halgren et al., 2002; Nobre and McCarthy, 1995), and it is therefore important to test whether it is already activated at earlier latencies. We therefore defined a region-of-interest (ROI) based on the peak of activation in the left mid-anterior temporal lobe for the average of words and pseudowords, which was then used to test for a significant difference between words and pseudowords. Spatial resolution of EEG and MEG is inherently limited (Hauk et al., 2011; Molins et al., 2008), but a meaningful pattern of source estimates for early brain responses would strengthen the argument in favor of their theoretical significance.

**Fig. 6:**
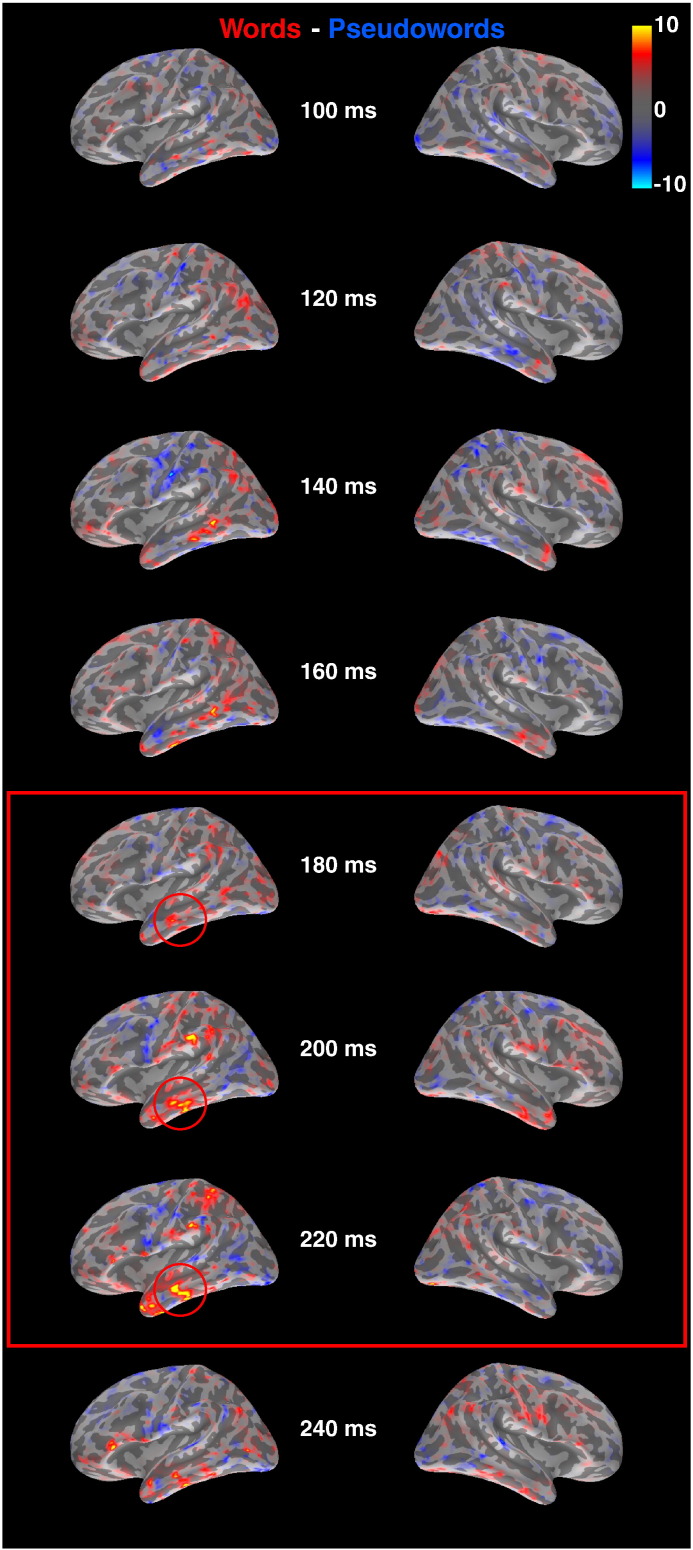
Estimates of the neuronal generators distinguishing words from pseudowords over time, derived from combined EEG and MEG measurements in Experiment 2. The difference of estimated neuronal activation for words and pseudowords was displayed on the inflated average cortical surface across subjects, separately for the left (left) and right (right) hemispheres. Red color indicates larger activation to words, blue color more activation to pseudowords. Units are in pA.

We estimated the neuronal generators of combined EEG + MEG data for words and pseudowords using distributed source analysis (minimum norm estimation, MNE). We did not have specific predictions about the generators of the living vs. non-living difference, because both categories were very broadly defined. Thus, we focused on the word-pseudoword difference in the lexical decision task, which is the most commonly used in research on visual word recognition. The difference of the resulting source distributions are shown in snapshots between 100 and 300 ms in Fig. 6. Red color indicates more activation for words compared to pseudowords. Left-lateralized activity in the temporal lobes becomes apparent around 200 ms. In the time window 180–220 ms, activity in the left anterior middle and inferior temporal lobes significantly differed between words and pseudowords (t(17) = 3.07, p < 0.01). It was also significant for the sub-windows 180–200 and 200–220 ms (t(17) = 3.12, p < 0.01 and t(17) = 2.80, p < 0.05, respectively). The corresponding effects in the right hemisphere were not significant (0 < t(17) < 1.0). In Fig. 4, there appears to be activity in the left inferior parietal lobe as well. However, this was not significant in the specified time windows (0 < t(17) < 1.1).

## Discussion

In the two experiments presented in this paper, we attempted to pose constraints on the absolute and relative timing of lexical and semantic information retrieval for single words. We collected behavioral and brain responses in a lexical and semantic Go/NoGo task, following the logic of previous studies on object recognition (e.g. Thorpe et al., 1996; VanRullen and Thorpe, 2001). We analyzed the fastest behavioral responses, and compared those to brain responses measured using ERPs and source estimates computed from combined EEG and MEG data. In both lexical and semantic tasks, the earliest behavioral responses distinguishing lexical and semantic stimulus categories occurred around 310 ms, and the corresponding ERP responses began to diverge around 160 ms. Combined EEG/MEG source estimation revealed more activation to words compared to pseudowords in the left anterior middle temporal lobe at 180 ms. Our results highlight the importance of combining fast behavioral responses and electrophysiology in studies on cognition and language.

We used a Go/NoGo paradigm, because it provides behavioral data as well as divergence points between response/no-response conditions in electrophysiological data (Thorpe et al., 1996). In behavioral studies on word recognition, it has been shown to yield similar patterns of results than the more conventional yes/no paradigm (Gomez et al., 2007). Furthermore, it allowed us to use eye blinks as a novel behavioral response type, in addition to conventional button presses, which resulted in shorter reaction times. We assumed that response preparation and execution account for about 150 ms in response times (Rayner, 1998; Thorpe et al., 1996). Thus, subtracting this latency from the earliest sensitive response times provides an estimate of the latency at which the brain has retrieved category-specific information (e.g., lexical information for words vs. pseudowords, and semantic information for living vs non-living things). Eye-blink responses to different word types demonstrated the earliest stimulus-sensitive behavioral responses at around 310 ms after word presentation. This is consistent with the finding that in natural reading fixations take about 200–300 ms, with a standard deviation of about 70 ms, and are already affected by lexical variables such as the lexical frequency of the fixated word (Kliegl et al., 2006; Rayner, 1998; Staub et al., 2010). This indicates that some lexical and semantic information must have been processed in this latency range. In our button press tasks, the earliest stimulus-sensitive response occurred around 400 ms, i.e., later than for eye blinks, which is consistent with a previous analysis of lexical decision data (e.g. Wagenmakers et al., 2008).

Our behavioral results demonstrated that eye blinks allow faster response times than button presses, and that they exhibit less variability. Thus, they may be more sensitive to subtle behavioral effects than conventional button presses. Eye blink responses may therefore be a useful methodology for researchers who focus on fast behavioral responses. Obvious limitations of this method are that 1) the speed of eye blinks is probably only an advantage in Go/NoGo paradigms, because eye blinks are usually performed with both eye lids simultaneously. The use of blinks for eyes individually (e.g. in “yes/no” tasks) still needs to be explored. 2) Eye blinks contaminate the EEG/MEG signal. Although effective artifact correction methods exist (Dimigen et al., 2011; Ille et al., 2002), signals occurring after the earliest eye blink latencies should be interpreted with care.

Our interpretation of the behavioral data is supported by our electrophysiological results. The earliest divergence between ERP signals in trials that required a response (“Go”) and those that did not (“NoGo”) occurred around 160 ms after stimulus presentation, both for lexical and semantic decisions. The information required to make this response must therefore be processed by the brain around that latency. The latency of 160 ms is remarkably similar to the one obtained by Thorpe and colleagues in their experiments on object categorization (Thorpe et al., 1996; VanRullen and Thorpe, 2001). Printed words can be considered as a special type of object. Nevertheless, they differ from real world objects in several important ways, e.g., with respect to spatial frequency, texture/color, fixation strategy, perceptual environment etc. It remains for future research to determine whether word recognition can be described by the same mechanisms as object processing, for example a fast feed-forward sweep through the visual system followed by recurrent activation (Lamme & Roelfsema, 2000; Riesenhuber & Poggio, 2002).

In a typical laboratory task it can take participants on average 600 ms or more to indicate by button press whether a visually presented letter string (such as “neuron” or “nouren”) is a word or not (e.g. Balota, Cortese, Sergent-Marshall, Spieler, & Yap, 2004). This begs the question of how we can account for the time between these early categorization processes and these late average response times, especially if our estimate of 150 ms for response preparation and execution is accurate. The earliest responses reflect – by definition – only a small proportion of all responses. What does it mean, then, that a small proportion of stimuli produce response times between 300 and 400 ms? Our Tables 2 and 3 demonstrate that these responses are not due to untypical stimulus features, suggesting that they are representative of the stimulus category. The challenge for the supporters of “late” (i.e., in the N400 range) lexical and semantic processing then is: What does a brain response between 300 and 500 ms tell us when a significant amount of behavioral responses already occur in this latency range? The supporters of “early” (i.e., pre-200-ms) lexico-semantic processes face the question: What do brain responses before 200 ms tell us when the largest proportion of behavioral responses occurs after 400 ms? The answer to these questions depends on where we assume the major source of variability of response times. In one extreme, we may assume the early word recognition processes to be constant, and the variability to originate from later evaluation or decision processes. In the other extreme, we may assume the word recognition process to be highly variable, and the decision phase to be constant.

In our view, there is good reason to assume that the early processes contribute less to variability than the later ones. Word recognition in skilled readers is a highly over-learned process. Recognizing words in reading is a hundreds-of-times-a-day task, while performing lexical or semantic decisions in a psychophysiological laboratory is for many a “once-in-a-lifetime” experience. Word recognition is often described as automatic, for example because most stages of word recognition are susceptible to masked priming (Dehaene et al., 2004; Grainger and Holcomb, 2009; Kiefer and Martens, 2010; Kinoshita and Quinn, 2008; McNamara, 2005; Neely, 1991). This corresponds to the more general finding that unconscious perception of visual stimuli modulates brain responses up to about 200 ms, while effects of conscious perception are reflected after 200–300 ms (see e.g. Dehaene and Changeux, 2011). These data indicate that early word recognition processes are relatively stable. Consistent with this view, the difference in reaction time distributions between lexical decision tasks with different decision criteria has been explained by the boundary separation variable in the Ratcliff diffusion model, rather than diffusion rate or dead time (Wagenmakers et al., 2008). Furthermore, as already described in the Introduction, fixation durations in natural reading are affected by lexical and context variables (Kliegl et al., 2006; Rayner, 2009). It has been shown that word frequency effects on fixation durations are not just due to exceptionally long fixations, but affect the whole distribution (Staub et al., 2010). Reaction time distributions in our button press tasks ranged from about 400 ms to 1500 ms. For fixation durations, they are commonly within 500 ms (Dimigen et al., 2011; Staub et al., 2010). The onset of the N400 component in sentence reading has been estimated to occur after the end of a fixation duration for the majority of trials (Dimigen et al., 2011). The fact that the topographies of ERP responses to different psycholinguistic variables in the N400 time range are very similar has been taken as evidence that they do not reflect processes related to specific types of word information, but rather integration processes in large-scale networks (Hauk et al., 2006a, 2006b). Our present data support this view, and provide further evidence that essential processes of lexical and semantic information retrieval occur within the first 200 ms of word onset.

Our behavioral data did not provide reliable evidence for serial retrieval of lexical and semantic information. Mean reaction times differed slightly between lexical and semantic decision in Experiment 2 (eye blinks), but the earliest response latencies appeared to be similar. It is therefore possible that the difference in mean reaction times is not due to a shift of the whole reaction time distribution, but only a subset of responses, e.g., only late ones. Therefore, we decomposed our reaction time distributions using Ex-Gaussian functions, which provide a better fit to positively skewed distributions than Gaussians that implicitly underlie any analysis using means or standard deviations (Lacouture and Cousineau, 2008). The parameters obtained from an Ex-Gaussian analysis cannot easily be mapped to specific cognitive processes, but they allow a more fine-grained comparison of reaction time distributions than conventional analyses (Matzke and Wagenmakers, 2009). None of the three Ex-Gaussian parameters in our analysis differed between tasks. Of course, we cannot conclude from the absence of these effects that the latencies for lexical and semantic processing are exactly the same, and therefore strictly parallel. However, we conclude that readers start to retrieve basic semantic information from letter strings at least almost as soon as they recognize a letter string as a word. This is consistent with connectionist models of word recognition which assume direct links between orthography and semantics (Rogers et al., 2004). Our findings raise the question of whether it makes sense to search for lexical and semantic effects in ERP data using separate components or time windows. A recent study has argued that both lexical and semantic processes occur in parallel within the N400 time window (Laszlo & Federmeier, 2010). Our results support near-simultaneous processing of lexical and semantic information, but indicate that they may start significantly earlier, already before 200 ms.

We analyzed brain activation for the lexicality effect around 200 ms in more detail using combined EEG and MEG measurements to estimate the neuronal sources of this activity. We only focused on the difference between words and pseudowords because our semantic categories (living and non-living) were too broadly defined to allow precise predictions of activation patterns. Significantly different levels of activation between words and pseudowords occurred in left anterior middle temporal cortex. Most neuroimaging studies comparing words and pseudowords have reported greater brain activation to the latter, although more activation for words compared to pseudowords has been interpreted as reflecting lexico-semantic retrieval (e.g. Jobard et al., 2003). According to the review of Jobard et al. (2003), this is most consistently found in basal and posterior middle temporal areas. Vinckier et al. (2007) reported a gradient from simple-to-complex word features along the posterior–anterior dimension of the inferior temporal lobe (Vinckier et al., 2007). The most anterior parts of this gradient, which activated most to words, may correspond to the activation observed in our study. Several authors have suggested that anterior temporal cortex is involved in general semantic processing, although there is still debate about the role of specific areas within this region (e.g. Binder et al., 2009; Moss et al., 2005; Patterson et al., 2007). In their meta-analysis, Binder et al. (2009) found the densest concentration of activation foci associated with semantic contrasts around the angular gyrus. We would like to note that we observed an activation focus compatible with this area around 200 ms (Fig. 6), but it did not reach significance. The spatial resolution of EEG and MEG data is inherently limited, and without restrictive modeling constraints at least in the range of several centimeters (Hauk et al., 2011; Molins et al., 2008). A more precise anatomical localization of this activation would therefore be speculative. In our view, in comparison to metabolic imaging methods the higher functional resolution of EEG/MEG due to timing information outweighs their lack of spatial precision. The roles of different brain areas in early lexico-semantic processing should be investigated in more detail by future studies using more specific psycholinguistic contrasts and task manipulations. Our results demonstrate that combined EEG and MEG are sensitive to early stimulus-specific processes, and provide further evidence that the brain accumulates lexico-semantic information about the stimulus already before 200 ms.

Our results have important implications for related areas of research. In electrophysiological research on emotion words, it is currently debated whether emotional valence affects brain responses at a lexical or post-lexical level (Kissler et al., 2009; Schacht and Sommer, 2009; Scott et al., 2009). In studies on the morphological structure of written words, it is still unclear how processing differs between regularly and irregularly structured words, and in particular whether regularly structured words are first decomposed into different morphemes and then re-integrated to compute the meaning (Marslen-Wilson and Tyler, 2007; Taft and Forster, 1975). Recent ERP and ERF studies have attempted to track the time course of these processes, with partly inconsistent results (Lavric et al., 2007; Lehtonen et al., 2007; Morris et al., 2007; Munte et al., 1999; Zweig and Pylkkanen, 2009). Our experimental approach would be applicable in those research areas as well, and our results on lexical and basic semantic processing may guide analysis strategies in future studies.

Based on behavioral data and computational considerations, some authors have already pointed out that there are no “magic moments” in word recognition, where parcels of information are unpacked and the contents handed over to the next stage (Balota and Yap, 2006). Our results support the view that word recognition should be thought of as continuous accumulation of evidence, rather than a sequence of stages. It has been demonstrated that the assumption that word recognition is Bayesian decision making process can explain a range of behavioral phenomena (Norris, 2006). According to our data, evidence is accumulated at a remarkable speed, comparable to previously reported data for object recognition. In how far computational models of object and word processing that have been designed in the behavioral domain can also be applied to electrophysiological data remains one of the most exciting challenges for the future. Constraining the time course of visual word recognition linking behavioral and electrophysiological data is an important step in this direction. The development of more sensitive tools to study weak early effects in more detail, for example using parametric or multi-variate techniques (Amsel, 2011; Chan, Halgren, Marinkovic, & Cash, 2011; Dien, Frishkoff, Cerbone, & Tucker, 2003; Hauk et al., 2006a, 2006b; Laszlo and Federmeier, 2010), should therefore have high priority in future research.

## Figures and Tables

**Table 1:** Stimulus properties.

	Letter length	Word frequency	Lemma frequency	Positional bigram	Positional trigram	Neighborhood size
Words LD	5.2	10.3	18.2	34,512	3824	3.4
Pseudowords LD	5.1	NA	NA	35,054	3504	3.0
Living SD	5.2	8.7	16.7	34,381	4072	4.2
Non-living SD	5.4	10.2	18.7	33,947	3982	4.6

Psycholinguistic parameters for all stimulus categories. Frequencies are reported as per million (note there are no word frequencies for pseudowords). LD: lexical decision; SD: semantic decision.

**Table 2:** Stimulus ranking with RT, lexical decision.

	S1	S2	S3	S4	S5	S6	S7	S8	S9	S10	S11	S12	S13	S14
1	Sloun	Slogan	Carpet	Farve	Basket	Glue	Frang	Gosling	Losh	Gidge	Knee	Foy	Cauth	Thimble
2	Plift	Fountain	Primate	Morsh	Myth	Hammer	Durve	Gym	Churm	Conge	Margin	Ralve	Plift	Basket
3	Ginse	Towel	Owl	Prute	Powder	Beacon	Turl	Womb	Drong	Bremp	Anchor	Drosh	Sniesubs	Gym
4	Flait	Garlic	Spur	Durve	Omelette	Fuel	Drong	Chest	Woir	Turl	Brewer	Harge	Slurt	Mantle
5	Dawth	Weasel	Hostel	Maft	Button	South	Gleigh	Kidney	Skalt	Narch	Castle	Craff	Maft	Arch
6	Drook	Verb	Canyon	Bremp	Raccoon	Liquor	Narty	Towel	Quish	Yall	Towel	Dawth	Ploist	Chapel
7	Tredge	Mantle	Powder	Colch	Bride	Vest	Dince	Chain	Nift	Avol	Rifle	Tarve	Screal	Chest
8	Vursk	Heap	Banner	Nimp	Glue	Bulb	Gruck	Furrow	Scrend	Nift	Oat	Ploist	Chount	Primate
9	Whike	Canyon	Oven	Shonk	Squad	Radish	Cradth	Wasp	Toove	Jing	Sky	Nift	Dawth	Plank
10	Tarve	Arch	Granite	Fouch	Ass	Canyon	Glish	Globe	Craff	Tanch	Prize	Farve	Woir	Beacon
Last-9	Gidge	Farce	Abode	Chark	Thimble	Comb	Blean	Mammoth	Tanch	Brout	Cork	Arge	Strimp	Costume
Last-8	Nift	Gong	Tempest	Tymposm	Limb	Guava	Brate	Clam	Plown	Smean	Finch	Blice	Briss	Arc
Last-7	Pserence	Prank	Jot	Vern	Knot	Rook	Blorn	Margin	Dulge	Blisk	Cosmos	Premp	Strick	Louse
Last-6	Skipe	Nil	Myth	Plounce	Slang	Skunk	Tanch	Raid	Pserence	Swook	Thimble	Sturp	Nizz	Rook
Last-5	Verm	Mime	Booth	Chisk	Chive	Spur	Prace	Ostrich	Swook	Doint	Shrub	Prace	Cremp	Leash
Last-4	Poy	Ass	Grain	Sniesubs	Margin	Robot	Fouch	Poppy	Prace	Marce	Gull	Glump	Smirch	Symptom
Last-3	Toove	Chimp	Skunk	Malge	Swine	Shrew	Ginse	Grub	Larp	Meague	Tempest	Plown	Ginse	Mime
Last-2	Tymposm	Silver	Clot	Skipe	Louse	Hash	Clarp	Scene	Frent	Stive	Radish	Dolve	Blice	Crown
Last-1	Clice	Salad	Fighter	Glump	Gull	Chimp	Arge	Barge	Preat	Fance	Abode	Corve	Glist	Gosling
Last	Blinch	Barge	Ass	Ploist	Prank	Raccoon	Brell	Canyon	Tymposm	Traut	Raid	Caintens	Mish	Brewer

Stimuli that were responded to fastest (top) or slowest (bottom) in the lexical decision task of Experiment 1, for all participants separately. Only hits are shown. Note that half of the participants had to respond to words, half to pseudowords.

**Table 3:** Stimulus ranking with RT, semantic decision.

	S1	S2	S3	S4	S5	S6	S7	S8	S9	S10	S11	S12	S13	S14
1	Arrow	Goose	Snail	Blanket	Germ	Rabbit	Spear	Spider	Braids	Buckle	Peach	Spanner	Piano	Mushroom
2	Chisel	Carrot	Mango	Thread	Wombat	Corn	Bread	Bone	Lamp	Marbles	Pig	Cello	Folder	Zebra
3	Rake	Camel	Budgie	Shower	Walrus	Turtle	Pencil	Mouse	Carriage	Plate	Melon	Dagger	Pistol	Leopard
4	Tongs	Squirrel	Cherry	Blouse	Cactus	Antelope	Lamp	Robin	Sandal	Violin	Eel	Spear	Rake	Raven
5	Bullet	Banana	Nose	Cigar	Frog	Cabbage	Mattress	Elephant	Spoon	Blender	Pigeon	Battery	Spade	Dolphin
6	Blade	Monkey	Banana	Jar	Oyster	Monkey	Scooter	Grass	Cake	Drum	Ear	Jar	Violin	Birch
7	Ladle	Lobster	Lobster	Apron	Shrimp	Pony	Pillow	Apple	Rail	Ribbon	Bee	Cleaver	Banjo	Ivy
8	Basin	Gnat	Otter	Iron	Cucumber	Sheep	Bucket	Flea	Drawer	Crayon	Vine	Wallet	Apron	Rat
9	Spanner	Spinach	Eagle	Umbrella	Spider	Maple	Vase	Bean	Battery	Ink	Potato	Drill	Clock	Celery
10	Spear	Zebra	Berry	Ladder	Panda	Deer	Axe	Chicken	Lantern	Broom	Hawk	Jumper	Tank	Swan
Last-9	Braids	Opossum	Penguin	Violin	Barley	Clown	Cake	Belly	Pencil	Pencil	Fern	Tram	Volcano	Clown
Last-8	Quilt	Tonsil	Falcon	Compass	Shark	Budgie	Shawl	Hawk	Tongs	Harpoon	Sheep	Crayon	Quilt	Porpoise
Last-7	Volcano	Tapir	Sheep	Dagger	Bean	Cobra	Spool	Neck	Socket	Cleaver	Mallard	Overalls	Iron	Chicken
Last-6	Drain	Crow	Clown	Jet	Brain	Shark	Braids	Pine	Piano	Pump	Lark	Hose	Sock	Pollen
Last-5	Canoe	Oak	Dolphin	Telly	Baboon	Gnat	Stool	Scorpion	Log	Bomb	Parrot	Axe	Buckle	Fowl
Last-4	Folder	Corn	Gorilla	Scissors	Monkey	Egg	Tunic	Petal	Duster	Cord	Bean	Stove	Brooch	Barley
Last-3	Bread	Foal	Cobra	Cello	Bull	Daisy	Oar	Boar	Overalls	Vat	Wolf	Pyramid	Trowel	Onion
Last-2	Glacier	Budgie	Worm	Razor	Pepper	Onion	Scissors	Buzzard	Clippers	Pyramid	Opossum	Rail	Toaster	Corn
Last-1	Tunic	Elbow	Bush	Spoon	Wren	Camel	Stove	Panther	Glove	Apron	Buzzard	Torch	Menu	Cherry
Last	Toaster	Python	Starling	Canoe	Pollen	Leopard	Tablet	Pheasant	Bread	Volcano	Bug	Peg	Battery	Belly

Stimuli that were responded to fastest (top) or slowest (bottom) in the semantic decision task of Experiment 1, for all participants separately. Only hits are shown. Note that half of the participants had to respond to living things, half to non-living things.
